# Integration of Electrical Properties and Polarization Loss Modulation on Atomic Fe–N-RGO for Boosting Electromagnetic Wave Absorption

**DOI:** 10.1007/s40820-024-01518-x

**Published:** 2024-10-18

**Authors:** Kaili Zhang, Yuefeng Yan, Zhen Wang, Guansheng Ma, Dechang Jia, Xiaoxiao Huang, Yu Zhou

**Affiliations:** 1 State Key Laboratory of Precision Welding & Joining of Materials and Structures, Harbin Institute of Technology, Harbin, 150001 People’s Republic of China; 2 School of Materials Science and Engineering, Harbin Institute of Technology, Harbin, 150001 People’s Republic of China; 3 MIIT Key Laboratory of Advanced Structural-Functional Integration Materials & Green Manufacturing Technology, Harbin Institute of Technology, Harbin, 150001 People’s Republic of China

**Keywords:** Electromagnetic wave absorption, Fe–N-RGO, Dipole polarization, Conduction loss, Impedance matching

## Abstract

Developing effective strategies to regulate graphene's conduction loss and polarization has become a key to expanding its application in the electromagnetic wave absorption (EMWA) field. Based on the unique energy band structure of graphene, regulating its bandgap and electrical properties by introducing heteroatoms is considered a feasible solution. Herein, metal-nitrogen doping reduced graphene oxide (M–N-RGO) was prepared by embedding a series of single metal atoms M–N_4_ sites (M = Mn, Fe, Co, Ni, Cu, Zn, Nb, Cd, and Sn) in RGO using an N-coordination atom-assisted strategy. These composites had adjustable conductivity and polarization to optimize dielectric loss and impedance matching for efficient EMWA performance. The results showed that the minimum reflection loss (*RL*_min_) of Fe–N-RGO reaches − 74.05 dB (2.0 mm) and the maximum effective absorption bandwidth (EAB_max_) is 7.05 GHz (1.89 mm) even with a low filler loading of only 1 wt%. Combined with X-ray absorption spectra (XAFS), atomic force microscopy, and density functional theory calculation analysis, the Fe–N_4_ can be used as the polarization center to increase dipole polarization, interface polarization and defect-induced polarization due to d-p orbital hybridization and structural distortion. Moreover, electron migration within the Fe further leads to conduction loss, thereby synergistically promoting energy attenuation. This study demonstrates the effectiveness of metal-nitrogen doping in regulating the graphene′s dielectric properties, which provides an important basis for further investigation of the loss mechanism.

**Supplementary Information:**

The online version contains supplementary material available at 10.1007/s40820-024-01518-x.

## Highlights

- Single-atom Fe–N_4_ sites embedded into graphene were successfully synthesized to exert the dielectric properties of graphene.
- The absorption mechanisms of metal-nitrogen doping reduced graphene oxide mainly include enhanced dipole polarization, interface polarization, conduction loss and defect-induced polarization.
- Excellent reflection loss of − 74.05 dB (2.0 mm) and broad effective absorption bandwidth of 7.05 GHz (1.89 mm, with filler loading only 1 wt%) were obtained.

**Supplementary Information:**

The online version contains supplementary material available at 10.1007/s40820-024-01518-x.

## Graphical abstract

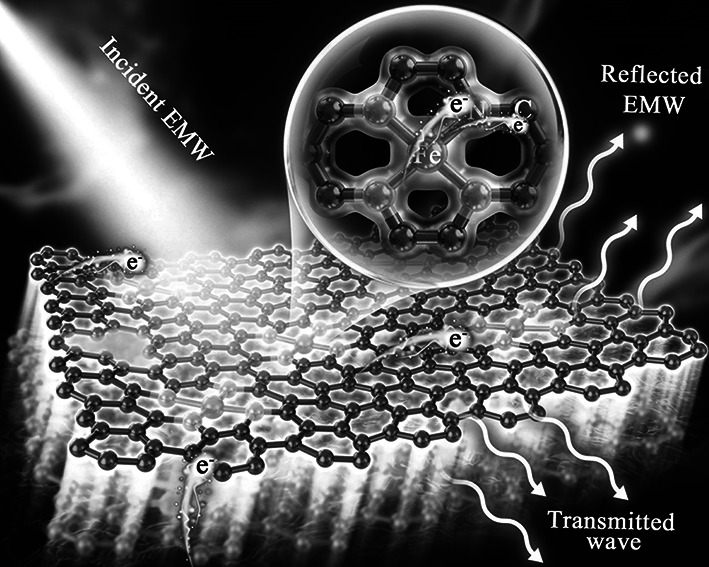

## Introduction

With the development of science and technology, electromagnetic radiation pollution is increasingly aggravated due to the wide application of electronic equipment [1–3]. Electromagnetic radiation not only interferes with the normal operation of equipment, but also endangers human health. Designing and manufacturing high performance electromagnetic wave (EMW) absorbing materials has become a research hotspot to protect electronic devices and humans from electromagnetic interference and radiation [4–6]. Carbon-based materials (such as graphite [7], carbon black [8], carbon nanotubes [9] and graphene [10]) have become a new type of electromagnetic wave absorption (EMWA) materials with great application potential due to their advantages of low density, large specific surface area, excellent conductivity, large dielectric loss and stable chemical properties.

As a two-dimensional carbon-based material with a single layer of carbon atoms, graphene has unique electronic properties and excellent electron transport ability, and has wide application prospects in the EMWA field [11–13]. However, the EMWA performance obtained directly from graphene is not ideal due to the contradiction between the graphene's filler loading, impedance matching degree and loss strength. Crystal phase transition, microstructure design, 3D conductive network, doping and defects are considered as effective strategies to adjust impedance matching and energy loss [14–19]. Recent studies have shown that it is difficult for graphene absorbent materials to achieve ideal EMWA at filling less than 5 wt% [20–22]. Therefore, achieving the co-optimization of impedance matching and high attenuation ability by regulating the electrical properties of graphene at low filler loading has become an important challenge in the EMWA field. So far, many strategies have been tried to regulate the conductivity of graphene, including vacancy [23], chemical doping [24], network structure [25], surface adsorption [26] and external field sources [27], etc. Considering the structural stability, process complexity and repeatability, heteroatom doping is undoubtedly an effective means to regulate the graphene′s electrical and dielectric properties..

Since the radius and extranuclear electron number of heteroatoms are different from those of carbon atom, heteroatom doping can affect the surface electronic structure and conductivity of carbon atoms to endow graphene materials more polarization sites [28]. Meanwhile, adjusting the morphology, defects and crystal phase of EMWA materials has been studied and employed to demonstrate the dielectric loss contribution through the introduction of dopants, which is also an effective strategy to reduce thickness and weight of materials [29–31]. Reasonable design of heteroatoms (such as O, N, F, S, and B) doped with graphene is an important means to open the bandgap of graphene. The type, location and density of heteroatoms can significantly modulate the electrical properties and dielectric loss mechanism of graphene. Liu et al. found that the N-bond configuration, conductivity, microstructure and dielectric properties of graphene could be precisely regulated by controlling the precursor concentration and reaction temperature. The result showed that the minimum reflection loss (*RL*_min_) is up to − 53.9 dB at 3.5 mm and the maximum effective absorption bandwidth (EAB_max_) is 4.56 GHz at 2 mm with a fill loading of 5 wt% [32]. The lattice defects and C–F bond polarization loss caused by F atoms in graphene oxide realized the EMWA of graphene absorbers in the S and X bands [33]. In addition, the diatomic (N/S and B/N) doping strategy can further broaden and modulate the bandgap and electrical properties of graphene and adjust the polarizability and conductivity by controlling heteroatoms content, so as to achieve the regulation of microwave absorption peak and absorption intensity [34–36]. The enhanced interfacial polarization effect and the dipole polarization caused by the binding of heteroatom and C atom as the polarization center play a key role in the EMW energy loss [37–39]. Doping studies of transition metal (Fe, Co, Ni) -graphene composites have been shown to be an effective way to alter the Fermi energy level distribution and increase the carrier concentration and mobility of graphene due to the large number of free electrons inside the metal [40–42]. But the electron injection amount in graphene is limited by the interface structure because the transition metal is only adsorbed on its surface. However, directly doping transition metal atoms in graphene network structures is difficult due to strong C–C covalent bonds and high energy barriers. We speculate that it is an effective method to doping transition metal atom coordination into graphene to form M–N–C composites by using the N atom as a "bridge", which is inspired by the atomically dispersed metal-nitrogen (M–N) catalyst strategy [43–45]. Establishing the connection between electrical, dielectric and EMWA properties is a key at the atomic scale in the EMWA field. Therefore, the stable anchoring of transition metal atoms on graphene is crucial for the preparation of lightweight graphene absorbers and also provides a new theoretical guidance for achieving "light, thin, wide and strong" EMWA properties.

In this work, we reasonably designed and synthesized a series of single metal atoms (M = Mn, Fe, Co, Ni, Cu, Zn, Nb, Cd, and Sn) doped reduced graphene oxide (M–N-RGO) with the assistance of N-coordination atoms, where M represents each metal atom. The doping of different metal atoms has a significant regulatory effect on the electrical properties of graphene. Taking Fe–N-RGO as an example, we investigated the modulation effect of Fe-N_4_ on the conductivity, permittivity and EMWA properties of RGO at the atomic scale and revealed the dielectric loss mechanism. Based on the X-ray absorption spectra (XAFS) results, electrical properties and density functional theory (DFT) calculation analysis, we certificated that the electric dipoles induced by the d-p orbitals hybridization between Fe, N, and C atoms cause dipole polarization. Meanwhile, the introduction of atoms caused structural distortion of graphene and increased its intrinsic defects, resulting in defect-induced polarization and enhanced interface polarization. In addition, graphene's lamellar network structure, large specific surface area and pore structure enhance conduction loss, interface polarization effect and multiple scattering. There is competition and synergy between the relaxation process caused by the defects, interfaces and conduction loss, resulting in the regulation of permittivity, which can effectively regulate the impedance matching of pure RGO to enhance the EMW energy loss. The results showed that the *RL*_min_ value of Fe–N-RGO is − 74.05 dB at 2.0 mm and the EAB_max_ achieves 7.05 GHz (1.89 mm) with a low filling loading only 1 wt%. The EMWA performance is significantly better than that of pure RGO, N-RGO, and Fe NPs/Fe–N-RGO, confirming that Fe and N atom co-doping can effectively regulate the dielectric polarization response and impedance matching of graphene. This designed strategy for atomic structure engineering can be a new pathway for the development of graphene absorbers.

## Experimental Section

### Materials

Urea (CO(NH_2_)_2_), FeCl_3_·6H_2_O and graphite powder (325 mesh and 99% purity) were purchased from Aladdin Co., ltd. (Shanghai, China). All chemicals were analytical grade (AR) without any further purification. The deionized (DI) water used was produced by the water purification system in the laboratory.

### Synthesis of M–N-RGO (M = Mn, Fe, Co, Ni, Cu, Zn, Nb, Cd, and Sn)

The GO was synthesized from natural graphite powder by the modified Hummers' method [5, 46]. The 0.5 g GO (5 mg mL^−1^, 100 mL) was sonication treated for 1 h. Then, 1.2 g CO(NH_2_)_2_ was added to the dispersion and magnetically stirred for 1 h. 10 mL FeCl_3_·6H_2_O aqueous solution (3 mg mL^−1^) was added to the mixture drop by drop and stirred for 2 h at room temperature. The mixture was frozen for 24 h until it was a solid sample in the refrigerator and treated by freeze-drying method (− 60 °C, 0.1 Pa) for 48 h. The above products were put into the tube furnace and carbonized at 800 °C for 1 h at a heating rate of 5 °C min^−1^ under Ar atmosphere. The as-obtained black solids were referred to as Fe–N-RGO. As comparison samples and the corresponding preparation conditions were consistent as above, the obtained samples were called RGO, Fe/RGO, and N-RGO in the absence of nitrogen and Fe sources. The obtained sample was named Fe NPs/ Fe–N-RGO under the condition of FeCl_3_·6H_2_O excessive addition (10 mL, 6 mg mL^−1^). In addition, the samples and synthesis process of M–N-RGO (M = Mn, Co, Ni, Cu, Zn, Nb, Cd, and Sn) were studied (Supporting Information). The M–N-RGO composites were similar to the Fe–N-RGO, except for the use of the corresponding metal salts instead of the iron salt.

### Characterization

The phase composition of the samples was characterized by X-ray diffraction (XRD) on a DX-2700 X-ray diffractometer. The morphology of the samples was obtained via scanning electron microscopy (SEM, ZEISS Merlin Compact). The diffraction patterns and high-resolution images were carried out using transmission electron microscopy (TEM, Talos F200X). The Fe content was determined by ICP-MS (NexION 350X, PerkinElmer). The high-angle annular dark field scanning transmission electron microscopy (HAADF STEM) images were recorded using a JEM ARM 200F scanning transmission electron microscope. The elemental composition of the samples was chartered by X-ray photoelectron spectroscopy (XPS, ESCALAB 250Xi). The Raman spectra were characterized by inVia-Reflex Raman spectroscopy system with 532 nm laser. The Fe K-edge XAFS were recorded on the BL14W1 beamline of the Shanghai Synchrotron Radiation Facility (SSRF) using a Si (111) double crystal monochromator. The functional groups on the surface of the samples were detected by Fourier transform infrared spectrometer (FTIR, Nicolet is50). The surface areas and pore structure of samples were measured by Brunner-Emmett-Teller (BET, BSD-PS) method and the corresponding Barret-Joyner-Halenda (BJH) method, respectively. The magnetic hysteresis loops of the material were tested by physical property measurement system (PPMS, Dynacool-14 T). The electrical properties were carried out by scanning probe microscope (SPM, Bruker, Dimension Fastscan). The coaxial shaped samples (Φin:3.04 mm, Φout:7.00 mm, d = 2.0 mm) were mixed with paraffin at a filler loading of 1 wt%. Electromagnetic parameters (ε_r_ and μ_r_) were measured using a vector network analyzer (VNA, Agilent N5245A). The RL values were calculated based on the transmission-line theory as follows [47, 48]:

1 $$\text{RL}(\text{dB})=20\text{ log}\left|\frac{{Z}_{in}-{Z}_{0}}{{Z}_{in}+{Z}_{0}}\right|$$

2 $${Z}_{in}={Z}_{0}\sqrt{\frac{{\mu }_{r}}{{\varepsilon }_{r}}}\text{tanh}\left(j\frac{2\pi fd}{c}\sqrt{{\mu }_{r}{\varepsilon }_{r}}\right)$$

where ${Z}_{in}$ represents the input impedance and ${Z}_{0}$ is the impedance of free space, *d* and *c* are thickness of the absorber and velocity of light.

### Density Functional Theory Calculation Details

All of the calculation results of graphene, N-graphene and M–N-Graphene (M = Mn, Fe, Co, Ni, Cu, Zn, Nb, Cd, and Sn) were carried out by the castep module of Materials Studio software based on DFT. The ion–electron interactions were described by the OTFG ultrasoft pseudopotential. The Generalized Gradient Approximation (GGA) method with Perdew-Burke-Ernzerhof (PBE) functional was adopted to solve the exchange and correlation functional energies. The cutoff energy and k-point mesh were determined as 800 eV and 5 × 5 × 1, respectively. The calculation accuracy was 1.0 × 10^−5^ eV per atom. The thickness of the vacuum layer along the c-axis is fixed at 15 Å.

## Results and Discussion

### Synthesis and Characterization of Fe–N-RGO

**Fig. 1:**
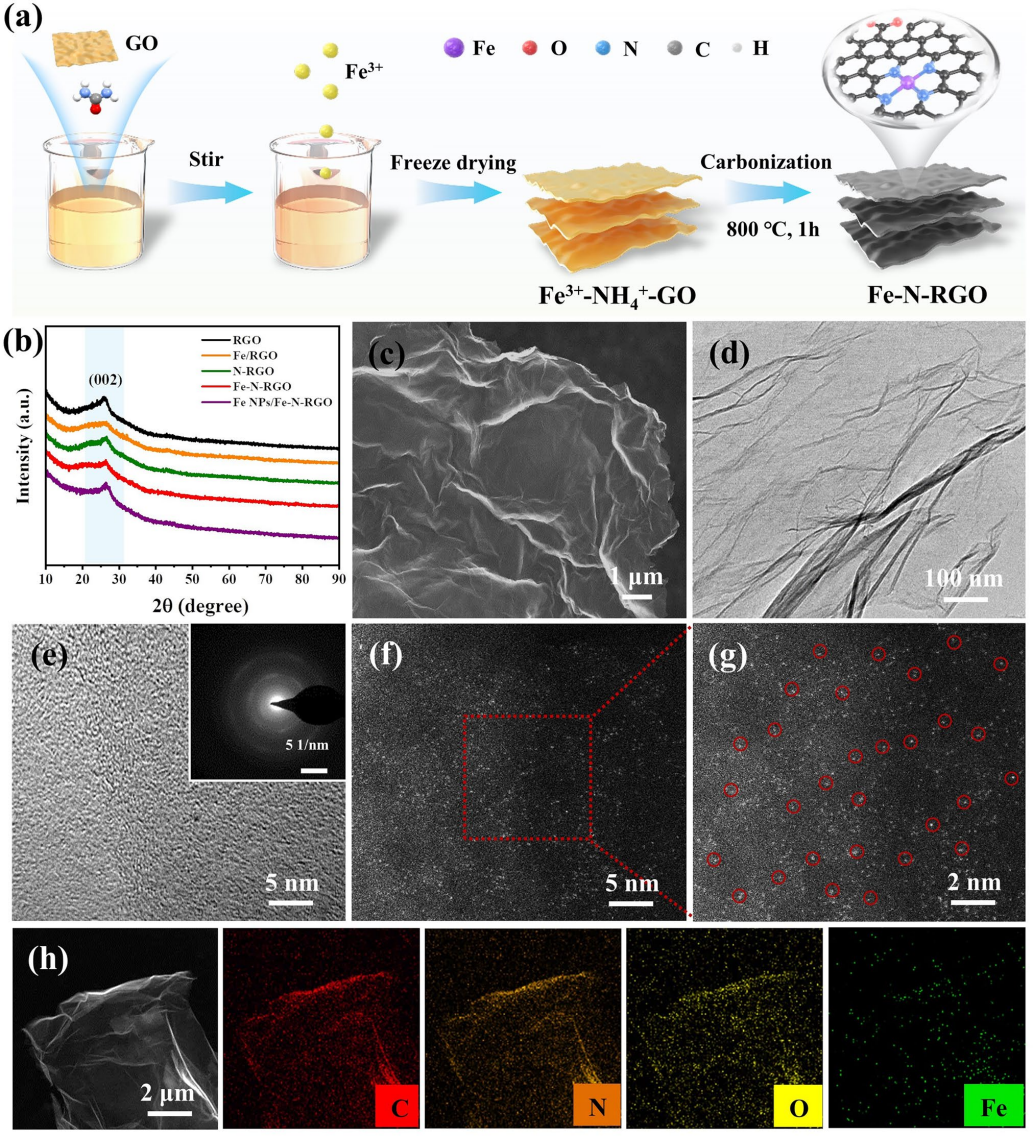
Preparation process and microstructural characteristics of Fe–N-RGO. **a** Schematic illustration of the preparation process of the Fe–N-RGO composite. **b** XRD patterns, **c** SEM image, **d** TEM image, **e** HRTEM images (inset: SAED pattern), **f, g** HAADF-STEM images and **h** STEM image and EDS elemental mappings of Fe–N-RGO

Fe–N-RGO was prepared by introducing urea as an N source and Fe^3+^ as a Fe source mixed with GO through simple freeze-drying and carbonization methods. The preparation process diagram of Fe–N-RGO is shown in Fig. 1a. The reference samples are labeled as RGO, Fe/RGO, N-RGO, and Fe NPs/Fe–N-RGO based on the presence of Fe and N dopants. The crystal structure of samples was characterized by XRD as shown in Fig. 1b, all samples have wide characteristic peaks at about 26.5°, corresponding to graphite (002) crystal plane. SEM and TEM results show that Fe–N-RGO exhibits a wrinkled sheet with no obvious nanoparticles, which is consistent with the microscopic morphology of RGO and N-RGO (Figs. 1c, d and S1a-e). HRTEM image and the SAED pattern results show that Fe–N-RGO has an amorphous structure and no visible Fe nanoparticles, indicating that Fe exists in the form of isolated atoms (Fig. 1e) [49]. The distribution of Fe–N-RGO on atomic scale was characterized using atomic resolution HAADF-STEM. Those isolated bright spots marked by red circles represent partially scattered Fe atoms because of the much larger atomic number than C. It can be observed that Fe atoms are uniformly dispersed in Fe–N-RGO (Fig. 1f, g). The elemental distribution of Fe–N-RGO is shown in Fig. 1h, in which the C, N, O, and Fe elements are uniformly dispersed in RGO. As a reference sample, Fe/RGO shows that Fe is uniformly dispersed on RGO with ~ 10 nm nanoparticles, indicating that the N atom plays an indispensable role of coordination atom to participate in the synthesis of Fe–N-RGO (Fig. S1c, f). From the TEM images as shown in Fig. S1g–i, with the increase of Fe sources, Fe nanoparticles (with 20 –50 nm) are loaded on RGO sheets. STEM-EDS element mapping results indicated that C, N, and O were uniformly distributed in RGO, while Fe was distributed on RGO and Fe nanoparticles in Fe NPs/ Fe–N-RGO. Moreover, ICP-MS analysis revealed Fe contents of 0.86, 2.6, and 3.3 wt% in Fe/RGO, Fe–N-RGO, and Fe-NPs/Fe–N-RGO (Fig. S2a).

**Fig. 2:**
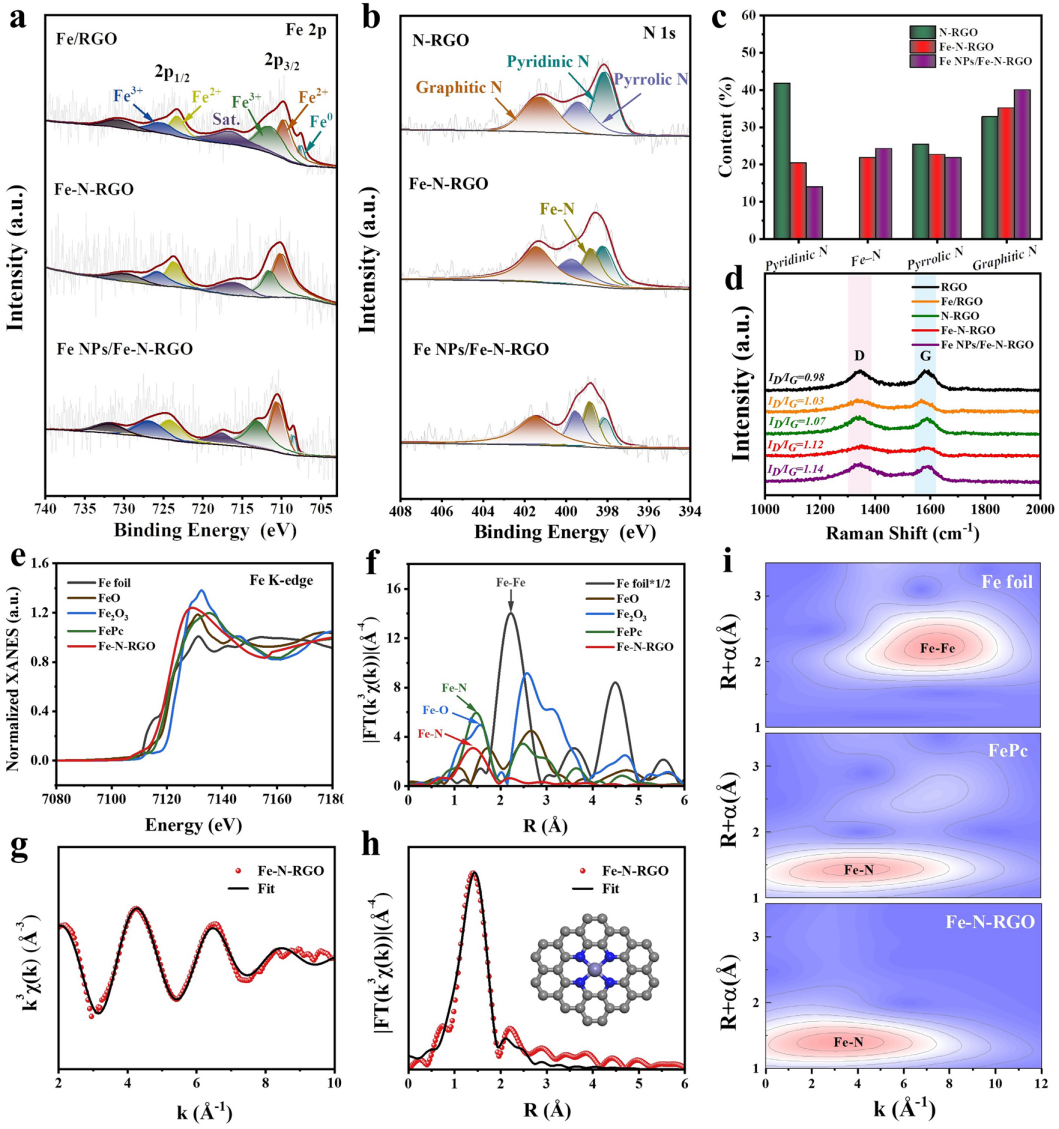
Atomic structure characterization of samples. The high-resolution **a** Fe 2*p*, **b** N 1*s* XPS spectrum and **c** the corresponding N content in samples. **d** Raman spectra. **e** Fe K-edge XANES spectra. **f** FT-EXAFS curve. EXAFS fitting curve at **g** k-space, **h** R space and the atomic structure model of Fe–N-RGO. **i** Wavelet transform contour plots of Fe foil, FePc and Fe–N-RGO

To analyze the atomic structure of Fe–N-RGO, XPS spectroscopy, Raman and XAFS of samples were characterized. The XPS full spectrum shows that Fe–N-RGO is mainly composed of Fe, O, N, and C, indicating that Fe and N are successfully doped into RGO (Fig. S2b). The high-resolution Fe 2*p* spectrum shows two typical peaks at 710.1 and 723.7 eV, belonging to Fe 2*p*_3/2_ and Fe 2*p*_1/2_ (Fig. 2a). It can be resolved into Fe^2+^ 2*p*_3/2_ (709.6 eV), Fe^3+^ 2*p*_3/2_ (711.7 eV), Fe^2+^ 2*p*_1/2_ (723.6 eV), and Fe^3+^ 2*p*_1/2_ (725.5 eV), proving that Fe^2+^ and Fe^3+^ exist in the sample. Compared with Fe–N-RGO, Fe/RGO, and Fe-NPs/Fe–N-RGO exhibit Fe^0^ characteristic peaks (708.4 eV) due to the presence of Fe nanoparticles [50]. As shown in Fig. 2b, c, the N 1*s* spectrum of Fe–N-RGO and Fe-NPs/Fe–N-RGO can be decomposed into pyridinic N (398.2 eV), Fe–N_*x*_ (398.8 eV), pyrrolic N (399.7 eV) and graphitic N (401.4 eV). N-RGO only has pyridinic N, pyrrolic N and graphitic N. Fe–N_*x*_ is formed due to the interaction between pyridinic N and isolated metal Fe atoms in RGO [51]. The results show that the introduction of Fe could promote the Fe–N configuration, which will be expected to be regarded as the polarization site in the dielectric loss mechanism. Moreover, due to the introduction of N, the high-resolution C 1*s* spectrum (Fig. S2c) shows four peaks of C–C, C–O, C–N, and O–C=O at 284.8, 285.2, 286.4, and 288.9 eV. The results of FTIR spectra of samples indicate that N may be introduced into the carbon network structure to form C−N bonds (Fig. S3). The above results indicated that the N atom has been successfully doped into the carbon lattice to form Fe–N–C. From the Raman spectrum (Fig. 2d), the characteristic peaks D band and G band represent carbon defects and graphitization at 1349 and 2800 cm^−1^, respectively [52]. The *I*_D_/*I*_G_ value of samples increased from 0.98 to 1.14, indicating that the introduction of N and Fe atoms can cause distortion in the carbon network to create more defects, which can serve as dipoles to increase dielectric loss [53].

To further investigate the atomic structure and chemical coordination environment of Fe, X-ray absorption near-edge structure (XANES) and X-ray absorption fine structure (EXAFS) analyses of Fe–N-RGO were shown at the Fe K-edge. As shown in Fig. 2e, the absorption edge of Fe is closer to FePc, indicating that Fe is positively charged in Fe–N-RGO. Notably, no additional peak was observed at 7110.5 eV, indicating a planar structure [54]. In addition, the bonding properties of Fe were analyzed by Fourier transform EXAFS (Fig. 2f). Fe–N-RGO does not show significant Fe–Fe interactions (2.23 Å), indicating that Fe is atomically dispersed, which is consistent with HAADF-STEM results. It also shows that a distinct peak (1.4 Å) corresponds to the Fe–N bond with coordination numbers of 3.9, indicating that four N atoms form a plane structure centered on the Fe atom (Table S1) [55]. The fitting results also clearly showed that the Fe center bonds with four N atoms in the plane to form Fe-N_4_ embedded in the graphene skeleton (Figs. 2g, h and S4). Furthermore, the EXAFS wavelet transform of Fe–N-RGO shows intensity maximums at k = 3.95 Å^−1^, which further supports the N coordination structure in Fe–N-RGO (Fig. 2i). From the above XPS and XAFS results, it can be confirmed that the O atom does not participate in the coordination, but exists in the form of C–O and C=O bonds. The N_2_ adsorption–desorption isotherms were used to attain porous features of N-RGO and Fe–N-RGO composites. As shown in Fig. S5, the adsorption–desorption curves exhibit that the two samples were of classical type IV, suggesting a mesoporous material characteristic [56]. The specific surface area and the pore size of Fe–N-RGO are 233.4 m^2^ g^−1^ and 7.1 nm which are higher than those of N-RGO (203.8 m^2^ g^−1^ and 6.2 nm). The above results indicate that the large specific surface area and more pores of graphene sheets will increase the propagation path of EMWs, which is conducive to impedance matching and multiple scattering to improve the attenuation ability.

### Analysis of Electromagnetic Parameters and EMWA Performance

**Fig. 3:**
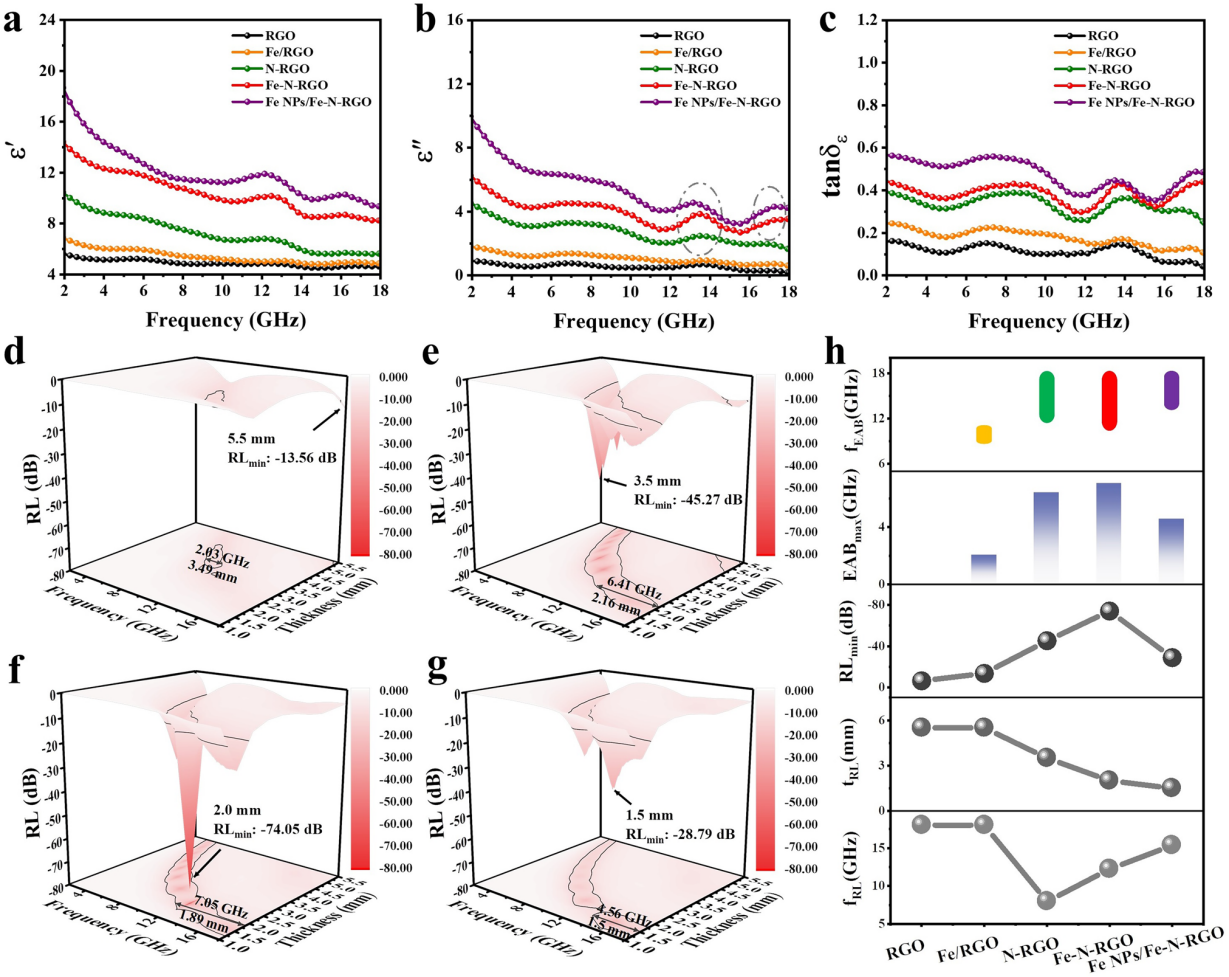
Analysis on electromagnetic parameters and EMWA performance of samples. **a** ε', **b** ε" and **c** tanδ_ε_. The 3D contour of RL values with different thickness and frequency of **d** Fe/RGO, **e** N-RGO, **f** Fe–N-RGO and **g** Fe NPs/Fe–N-RGO. **h** Summarization of EAB_max_, RL_min_ and the corresponding thickness of all the samples

In order to explore the absorbing properties, the electromagnetic parameters (ɛ_r_ = ɛ'-j·ɛ" and μ_r_ = μ'-j·μ") of samples (1 wt%) were characterized at 2–18 GHz. Figure 3a–c shows ε′, ε″, and tanδ_ε_ values of RGO, Fe/RGO, N-RGO, Fe–N-RGO, and Fe NPs/Fe–N-RGO. Pure RGO exhibits lower ε′ (5.62–4.52) and ε″ (0.89–0.26) values, which means that its dielectric loss plays a less role in EMWA due to low filling. Compared with the ${\varepsilon }_{r}$ of Fe/RGO, the dielectric response of N-RGO is greater in the electromagnetic field with ε′ of 10.38–5.62 and ε″ of 4.52–1.71. The introduction of N atoms could greatly increase the RGO defects, resulting in defect-induced polarization and interface polarization effect. Further, the introduction of Fe atoms intensifies the polarization loss in the Fe–N-RGO, and the ε′ and ε″ values are raised to 14.43–8.27 and 6.23–3.51. With the increase of Fe, the ε′ (18.57–9.37) and ε″ (9.74–4.32) of Fe NPs/Fe–N-RGO further increase due to defects polarization and the interface polarization between Fe nanoparticles and Fe–N-RGO. According to ε′ and ε″ curves, we can also obviously observe that N-RGO, Fe–N-RGO and Fe NPs/Fe–N-RGO all have polarization relaxation peaks, indicating that the dielectric loss caused by polarization loss has an important contribution to EMWA. Meanwhile, the complex permittivity of samples decreases with the increase in frequency, which is a typical dispersion behavior of carbon materials [57]. From the permeability curves (μ′ and μ″), it can be found that all samples have μ′ values close to 1 and μ″ values near 0, indicating that magnetic loss is negligible (Fig. S6). Figures 3d–g and S3 show the *RL* values of samples at different thicknesses and frequencies. As shown in Fig. S7, pure RGO shows poor EMWA performance. The introduction of Fe nanoparticles slightly increased the EMWA performance of Fe/RGO, where *RL*_min_ value is -13.56 dB at 18 GHz and EAB_max_ is 2.03 GHz (8.82–10.85 GHz) at 3.49 mm (Fig. 3d). Parallelly, the introduction of N atoms into RGO can significantly improve the EMWA performance, the N-RGO composite achieved *RL*_min_ of − 45.27 dB at 7.97 GHz and EAB_max_ of 6.41 GHz (11.59–18 GHz) at 2.16 mm (Fig. 3e). Fe and N atoms doped in RGO can further greatly improve the EMWA performance. The *RL*_min_ value of Fe–N-RGO reaches -74.05 dB at 12.22 GHz (2.0 mm) and the EAB_max_ achieves 7.05 GHz (10.95–18 GHz) at 1.89 mm (Fig. 3f). This is mainly due to the intrinsic defect-induced polarization, dipole polarization and strong interface polarization effect caused by atom doping. With the increase of Fe content, Fe NPs/Fe–N-RGO shows lower *RL*_min_ (− 28.79 dB, 15.35 GHz) and EAB_max_ (4.56 GHz, 13.44–18 GHz) at 1.5 mm (Fig. 3g). The carrier injection mechanism of Fe into RGO further enhances the interfacial polarization and conduction loss at the Fe/RGO interface, which is consistent with our previous work [58]. High conductivity can lead to strong reflection between air and material interface due to impedance mismatch, which limits further improvement in EMWA performance. More intuitively, the *RL*_min_ and the EAB_max_ values for all samples are summarized in Fig. 3h. Fe–N-RGO composite exhibits high loss and broadband EMWA performance, which can be interpreted as Fe–N–C synergistic dielectric polarization mechanism.

### Analysis of EMWA Loss Mechanism

To further elucidate the EMW dielectric loss mechanism, the attenuation mechanism of conductivity and polarization loss is analyzed. According to doping studies, both Fe and N can promote electron transport in the material to obtain good conductivity [59, 60]. Based on Debye theory, the ε_p_" and ε_c_" can be expressed as [61]:

3 $$ \varepsilon^{\prime } = \varepsilon_{\infty } + \frac{{\varepsilon_{s} - \varepsilon_{\infty } }}{{1 + \omega^{2} \tau^{2} }} $$

4 $$ \varepsilon^{\prime \prime } = \varepsilon_{p}^{\prime \prime } + \varepsilon_{c}^{\prime \prime } = \frac{{\varepsilon_{s} - \varepsilon_{\infty } }}{{1 + \omega^{2} \tau^{2} }}\omega \tau + \frac{\sigma }{{\omega \varepsilon_{0} }} $$

**Fig. 4:**
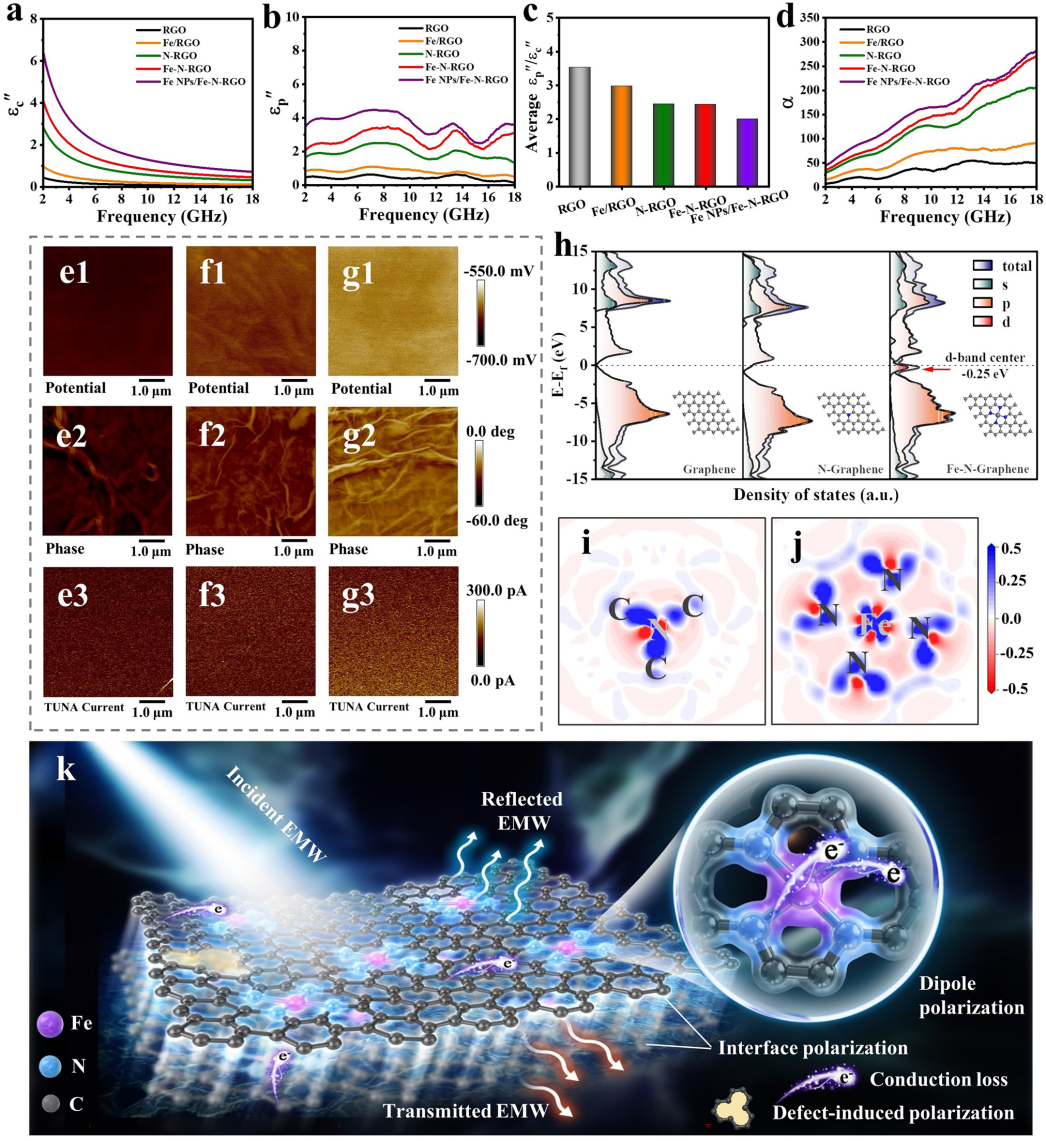
Analysis on EMWA mechanisms. **a** ε_c_″, **b** ε_p_″, **c** average ε_p_″/ε_c_″ and **d** α of RGO, Fe/RGO, N-RGO, Fe–N-RGO and Fe NPs/Fe–N-RGO. Surface potential, phase and current distribution images of **e** RGO, **f** N-RGO and **g** Fe–N-RGO. **h** DOS and PDOS results and illustration corresponding geometric structure model. Charge density difference diagrams of **i** N-Graphene and **j** Fe–N-Graphene (the blue and red represent electron accumulation and electron depletion). **k** EMWA schematic diagram of Fe–N-RGO

The ε_c_" and ε_p_" are the loss capacity of the conduction and polarization, respectively. As shown in Fig. 4a, b, the conductivity of Fe–N-RGO and Fe-NPs/Fe–N-RGO increases significantly due to the rapid transfer of electrons between Fe, N, and C, resulting in enhanced conduction loss (Fig. S8a). At the same time, the introduction of Fe and N increased the RGO defects resulting in defect-induced polarization. Further, orbital hybridization between Fe and N transfers electrons from Fe to N atoms, which leads to the formation of polarization sites for Fe–N bonds to enhance dipole polarization and interface polarization. The presence of Fe nanoparticles improves the conductivity but also introduces more heterogeneous interfaces, resulting in the simultaneous enhancement of conductivity loss, dipole polarization and interface polarization according to the carrier injection mechanism. Meanwhile, Fig. 4c shows that the average ε_p_"/ε_c_" values of the sample are higher than 1, indicating that the polarization loss is dominant. With the increase of Fe content, ε_p_"/ε_c_" gradually decreases due to the conductivity enhancement. It is widely believed that a high proportion of conduction loss is more likely to cause impedance mismatch. The attenuation constant (α) expresses the loss capability of EMW energy, which can be represented as [62]:

5 $$ \alpha = \frac{\sqrt 2 \pi f}{c} \times \sqrt {\left( {\mu^{\prime \prime } \varepsilon^{\prime \prime } - \mu^{\prime } \varepsilon^{\prime } } \right) + \sqrt {\left( {\mu^{\prime \prime } \varepsilon^{\prime \prime } - \mu^{\prime } \varepsilon^{\prime } } \right)^{2} + \left( {\mu^{\prime } \varepsilon^{\prime \prime } - \mu^{\prime \prime } \varepsilon^{\prime } } \right)^{2} } } $$

Figure 4d shows that the α of samples is gradually enhanced and increases with frequency. Polarization relaxation behavior is described by introducing Debye theory in this work. Each Cole–Cole semicircle represents a Debye relaxation process [63]. From Fig. S8b–f, the Cole–Cole curves of the samples have the same semicircle number indicating the same type of polarization relaxation. The smooth tails indicate that conduction loss is dominant at low frequencies, which is consistent with the results in Fig. 4a. In general, a good impedance match is also an important condition for absorbers [64]. However, the impedance mismatch caused by high conductivity is not conducive to energy loss (Fig. S9). Therefore, excellent EMWA performance to be obtained requires the synergetic effect of attenuation ability and impedance matching. As shown in Fig. S10, we can see that the *RL*_min_ values gradually shift toward to a low frequency with increasing the sample thickness, which can be explained by the quarter wavelength matching model [65–68]:

6 $$ t_{m} = \frac{n}{4}\lambda = \frac{nc}{{4f_{m} \sqrt {\left| {\mu_{r} \varepsilon_{r} } \right|} }}n = 1, 3, 5, \cdots $$

where ${t}_{m}$ is the matching thickness, ${f}_{m}$ is the matching frequency, $\lambda$ represent the wavelength of the EMW and ${{t}_{m}}^{exp}$ is the actual absorber thickness. When the matching thickness of the sample satisfies formula (6), incident wave will reflect 180° on each interface with inverse phases, leading to energy attenuation of EMW. For N-RGO and Fe–N-RGO, it is clear that these practical matching thicknesses are consistent with the simulated thicknesses, demonstrating that the superior EMWA performance obeys the quarter-wavelength matching model. Besides, ideal impedance matching values are desired with a |Z_in_/Z_0_| value of 1, which show that all incident EMW could enter the interior of the absorber for subsequent attenuation. The impedance matching values of N-RGO and Fe–N-RGO at different thicknesses are very close to 1, demonstrating efficient EMWA along with good impedance matching. Further, the electrical properties of RGO, N-RGO, and Fe–N-RGO at the micro-nano scale were characterized by atomic force microscopy (KPFM, EFM and C-AFM). Figure 4e1−e3 shows that the surface potential, phase and current distribution images of RGO are all in lower values (dark area). The electrical signal intensity of N-RGO and Fe–N-RGO are gradually increased and the color distribution are uniform (bright region) in Fig. 4f, g. The above results are mainly attributed to the following aspects: Firstly, the surface potential difference proves that the Fermi energy level of graphene has changed due to the doping of Fe atoms. Secondly, doping of Fe and N atoms forms strong electric dipoles (N–C and Fe–N–C), which enhances the internal potential and charge accumulation, resulting in a higher surface charge distribution. Thirdly, the surface current distribution images show an increasing trend due to electron migration of dopants to graphene, which enhances its conductivity.

To better understand the mechanism of doping, the atomic scale Fe–N–C structure was constructed based on XAFS and the charge density difference was calculated using DFT. As shown in the density of states (DOS) and partial density of states (PDOS) diagrams (Fig. 4h), the DOS of Fe–N-RGO is significantly higher than that of RGO and N-RGO at the Fermi level, and the center of the d-band is close to the Fermi level. This is due to the fact that Fe occupies more orbitals and electron distribution, which changes the graphene Fermi energy level and endows it richer electronic states. As shown in Fig. 4i, j, the blue and red regions represent electron accumulation and electron depletion. Obviously, the charge density difference results show that electron migration from the Fe atom to the N atom due to the 3*d*-2*p* orbital hybridization at the Fermi level, resulting in the charge being redistributed in Fe–N–C. Each Fe–N–C site can be regarded as a dipole (polarization center) forming an enhanced dipole polarization loss and interface polarization effect under electromagnetic fields. Therefore, the Fe–N doped RGO microstructure EMW loss mechanism was analyzed as shown in Fig. 4k. Firstly, graphene's sheets network structure is conducive to electron migration and jumping, which greatly enhances its conduction loss. Secondly, the introduction of atoms caused structural distortion of graphene and increased its intrinsic defects, resulting in defect-induced polarization and interfacial polarization. Most importantly, electrons are transferred from the Fe to the N atom due to the 3*d*-2*p* orbital hybridization, resulting in an enhanced Fe–N bond. The dipoles formed by Fe–N–C increase a large number of polarization centers in RGO lamella structure, leading to enhanced dipole polarization loss and strong interface polarization effect. Moreover, electron migration also enhances the conductivity of RGO, resulting in increased conduction loss. Hence, Fe–N-RGO exhibits remarkable EMWA performance under the coordination of various loss mechanisms.

### Analysis of EMWA Performance and Mechanism of M–N-RGO

**Fig. 5:**
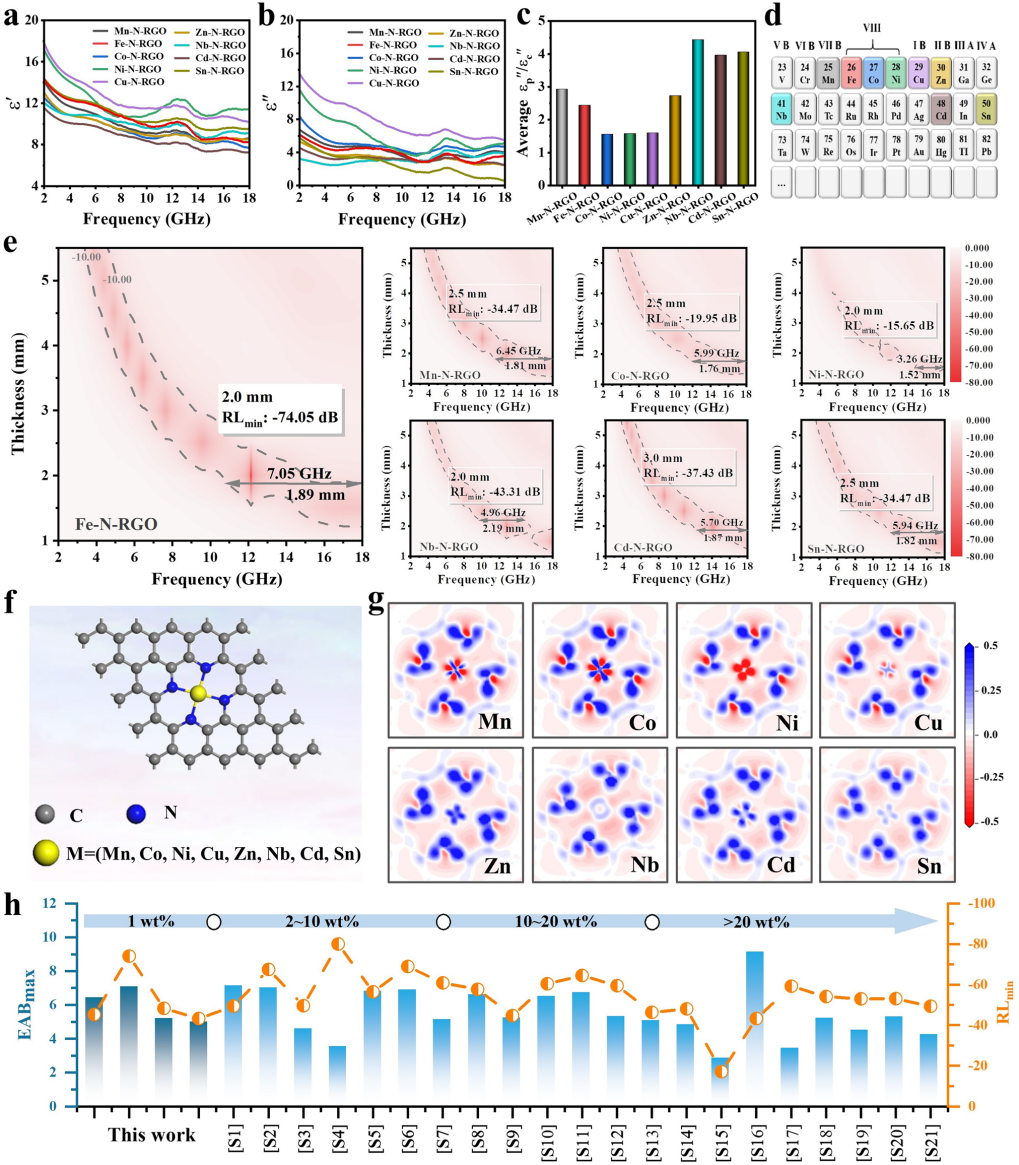
Analysis on EMWA performance of M–N-RGO. **a** ε', **b** ε", **c** average ε_p_″/ε_c_″and **d** partial periodic table of the elements. **e** 2D color-mapping *RL* values with different thickness of M–N-RGO composites. **f** Geometric structure model and **g** charge density difference plots of M–N-Graphene. **h** Comparison of EMWA performance in N-doped RGO-based materials

To further explore the dielectric loss mechanism induced by atomic doping, metal elements with physicochemical properties close to Fe were doped into RGO to obtain M–N-RGO composites (M = Mn, Fe, Co, Ni, Cu, Zn, Nb, Cd, and Sn). Their electromagnetic parameters (1 wt%) were measured, as shown in Fig. 5a-d. Interestingly, the ε′ and ε″ values of M–N-RGO are significantly different, while the μ′ and μ″ values of the permeability are ≈ 1 and ≈ 0, respectively (Fig. S11a–c). The obvious variation in dielectric properties of M–N-RGO may be attributed to the difference in the electrical properties of M–N. Figure 5c shows that the average ε_p_″/ε_c_″ values of M–N-RGO are higher than 1, indicating that polarization loss is dominant. This shows that the degree of polarization relaxation caused by metal atom doping is higher than the conduction loss, which is consistent with the separate conduction and polarization loss (Fig. S11d–f). In detail, the doping of elements with the same period as Fe further enhances the conductivity of graphene, while the doping of elements with the fifth period is the opposite due to the difference in electron transfer ability in M–N. According to the transmission line principle, M–N-RGO composite′s EMWA performance is calculated, as shown in Figs. 5e and S12a, b. Among them, Fe–N-RGO shows excellent *RL*_min_ (− 74.05 dB, 12.22 GHz) at 2.0 mm and EAB_max_ (7.05 GHz, 10.95–18 GHz) at 1.89 mm. The *RL*_min_ value of Mn-N-RGO is − 34.47 dB at 10.10 GHz (2.5 mm), the EAB_max_ reaches 6.45 GHz (11.55–18 GHz) at 1.81 mm. The *RL*_min_ values of Co–N-RGO and Ni–N-RGO are − 19.95 at 10.24 GHz and − 15.65 dB at 11.78 GHz, the EAB_max_ are 5.99 GHz (12.01–18 GHz) and 3.26 GHz (14.74–18 GHz), respectively. The EMWA property of Cu–N-RGO is significantly lower than those of the above materials due to impedance mismatch (Fig. S12c). The M–N-RGO composites (M = Zn, Nb, Cd and Sn) obtain significant EMWA properties due to suitable impedance matching and attenuation characteristics (Fig. S12). Similarly, to establish the connection between conduction, polarization, and EMWA at the atomic scale, the M–N-Graphene geometric model is constructed using first principles and its DOS, PDOS, and charge density difference are calculated (Fig. 5f, g). From Fig. 5g, the electronegativity difference of M–N–C atoms changes electron cloud distribution and M–N bonds also exhibit different charge transfer degrees (blue and red areas represent charge accumulation and charge depletion, respectively). The metal atoms (Mn, Co, Ni and Cu) gradually transfer electrons to N and C, showing a strong charge transfer due to orbital hybridization between M 3*d* and N 2*p*. The strong 3*d*-2*p* orbital hybridization mode causes the M–N–C to form an electric dipole, inducing an enhanced dipole polarization loss and interface polarization in the electromagnetic field. On the other hand, electron migration also enhances the conductivity of RGO, resulting in increased conduction loss. However, compared with the strongly interacting *d*-*p* orbital hybridization, M–N (Zn, Nb, Cd, and Sn) mainly exhibits the weakly interacting *s*-*p* orbital hybridization at the Fermi level of graphene. Low electron transfer between M–N (Zn, Nb, Cd, and Sn) bonds leads to low conduction and polarization losses (Fig. S13). Thus, each M–N–C site can be regarded as a dipole polarization center, and the orbital hybridization within it causes conduction and polarization losses to be enhanced to varying degrees, thereby increasing EMWA. Figure 5h summarizes the best EMWA performance of M–N-RGO composites in this work compared to other references related to N-doped RGO-based materials at different filling contents (Table S2). The new Fe–N-RGO composite will be an ideal candidate for a new generation of lightweight, broadband and highly efficient EMWA materials.

## Conclusion

In summary, we successfully prepared Fe–N-RGO by embedding single-atom Fe-N_4_ sites into RGO and explored the dielectric attenuation mechanism to establish the relationship between conduction, polarization and EMWA at the atomic scale. The XPS and XAFS results demonstrated the strong interaction between Fe–N bonds. AFM and DFT calculation analysis showed that the 3*d*-2*p* orbital hybridization in Fe–N_4_ induces the electric dipole formation leading to dipole polarization and electron migration leads to conduction loss and relaxation enhancement. In addition, the introduction of heteroatoms causes the graphene lamellar structure distortion to defect-induced polarization and interface polarization, thereby synergistically promoting EMW energy loss. The results showed that *RL*_min_ of Fe–N-RGO reaches − 74.05 dB at 2.0 mm and the EAB_max_ achieves 7.05 GHz at 1.89 mm with a low filler loading of only 1 wt%. Further, experimental and theoretical calculations have also confirmed that a series of metal heteroatom doping (M–N-RGO) can provide an effective means to modulate the electrical properties of graphene, which provides an important reference for refining the graphene′s dielectric loss mechanism.

## Supplementary Information

Below is the link to the electronic supplementary material.

Additional experimental characterization such as SEM and TEM images, ICP measurement,XPS analysis, FTIR spectra, EXAFS fitting curves, EMWA performance, σ, Cole–Cole plots,and impedance match of RGO, Fe/RGO, N-RGO, Fe-N-RGO and Fe NPs/Fe-N-RGO, andelectromagnetic parameter, attenuation constant, impedance match, RL values, DOS andPDOS of M-N-RGO composites. (DOCX 1840 kb)
