# Supplementary material for: Integration of Electrical Properties and Polarization Loss Modulation on Atomic Fe–N-RGO for Boosting Electromagnetic Wave Absorption
Source: Nanomicro Lett. 2024 Oct 18;17:46. doi: 10.1007/s40820-024-01518-x (PMC11489363; doi:10.1007/s40820-024-01518-x)
Supplement: Supplementary file 1 — Additional experimental characterization such as SEM and TEM images, ICP measurement,XPS analysis, FTIR spectra, EXAFS fitting curves, EMWA performance, σ, Cole–Cole plots,and impedance match of RGO, Fe/RGO, N-RGO, Fe-N-RGO and Fe NPs/Fe-N-RGO, andelectromagnetic parameter, attenuation constant, impedance match, RL values, DOS andPDOS of M-N-RGO composites. (DOCX 1840 kb) [file 40820_2024_1518_MOESM1_ESM.docx]

Supporting Information for

**Integration of Electrical Properties and Polarization Loss Modulation on Atomic Fe-N-RGO for Boosting Electromagnetic Wave Absorption**

Kaili Zhang^1, 2, 3^, Yuefeng Yan^2^, Zhen Wang^2, *^, Guansheng Ma^2^, DeChang Jia^1, 2, 3^, Xiaoxiao Huang^1, 2, 3, *^, Yu Zhou^1, 2, 3^

^1^ State Key Laboratory of Precision Welding & Joining of Materials and Structures, Harbin Institute of Technology, Harbin 150001, P. R. China

^2^ School of Materials Science and Engineering, Harbin Institute of Technology, Harbin 150001, P. R. China

^3^ MIIT Key Laboratory of Advanced Structural-Functional Integration Materials & Green Manufacturing Technology, Harbin Institute of Technology, Harbin 150001, P. R. China

*Corresponding authors. E-mail: [swliza@hit.edu.cn](mailto:swliza@hit.edu.cn) (Xiaoxiao Huang); [hagongdawangzhen@163.com](mailto:hagongdawangzhen@163.com) (Zhen Wang)

**S1 Experimental Section**

**S1.1 Synthesis of M-N-RGO (M= Mn, Co, Ni, Cu, Zn, Nb, Cd and Sn)**

First of all, metal salts of the same molar ratio were added into the 10 mL deionized water and stirred till solid particle were completely dissolved, respectively. Then, 0.5 g GO (5 mg/mL, 100 mL) was sonication treated for 1 h, and 8 parts were treated in the same way. 1.2 g CO(NH_2_)_2_ was added to the dispersion and magnetically stirred for 1h. 10 mL metal salt solution was added to the mixture drop by drop separately and stirred for 2 h at room temperature. The mixture was frozen for 24 h in the refrigerator and treated by freeze-drying method (-60 °C, 0.1 Pa) for 48 h. The above products were put into the tube furnace and carbonized at 800 °C for 1 h at a heating rate of 5 °C /min under Ar atmosphere. The black solids were referred to as M-N-RGO (M= Mn, Co, Ni, Cu, Zn, Nb, Cd and Sn).

**
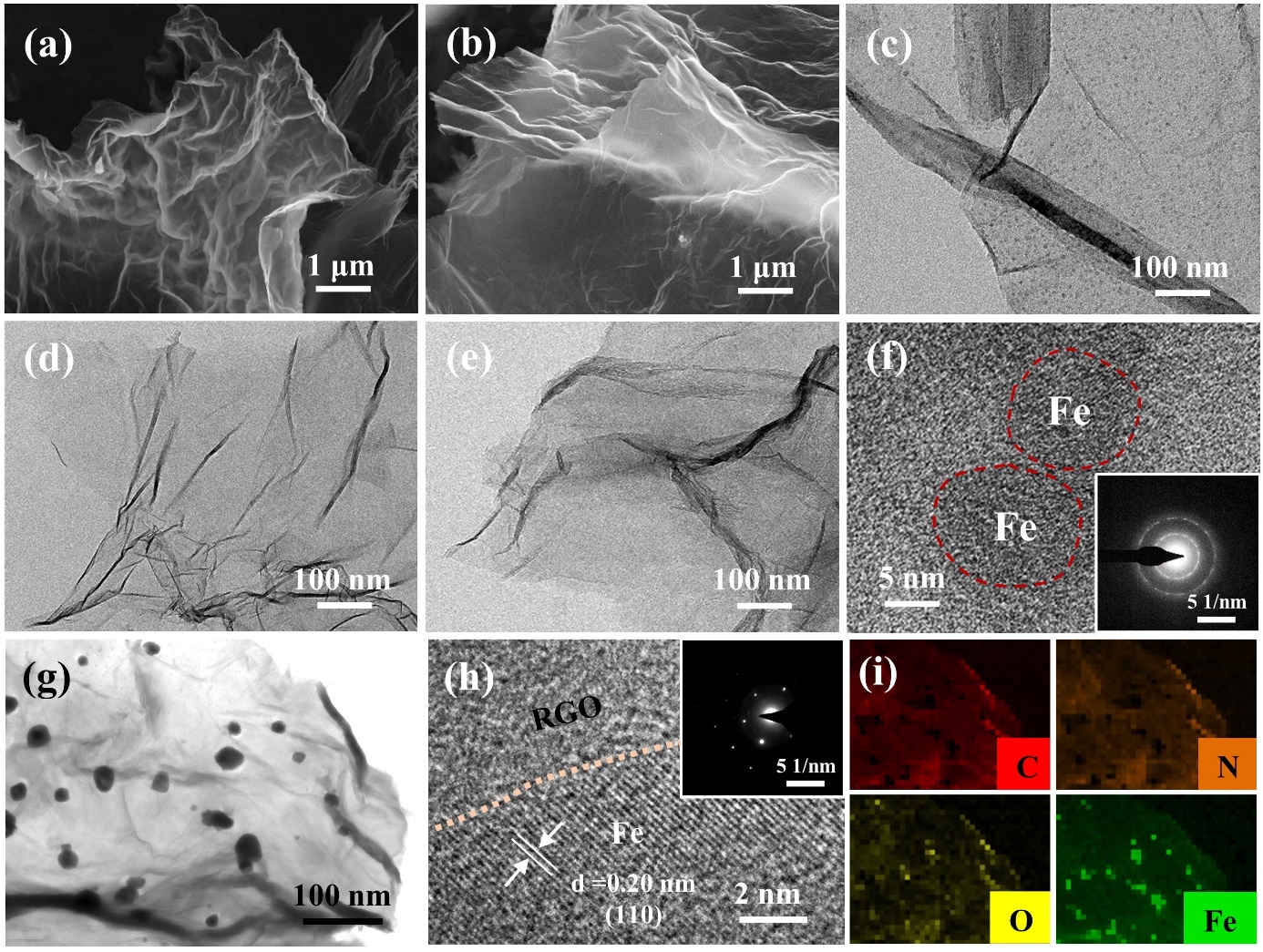
S2 Supplementary Figures and Tables**

**Fig. S1** Microstructural characterization of samples. **a, d** SEM and TEM images of RGO. **b, e** SEM and TEM images of N-RGO. **c, f** TEM images of Fe/RGO (inset: SAED pattern). **g, h, i** TEM, HRTEM images and EDS elemental mappings of Fe-NPS/Fe-N-RGO

**
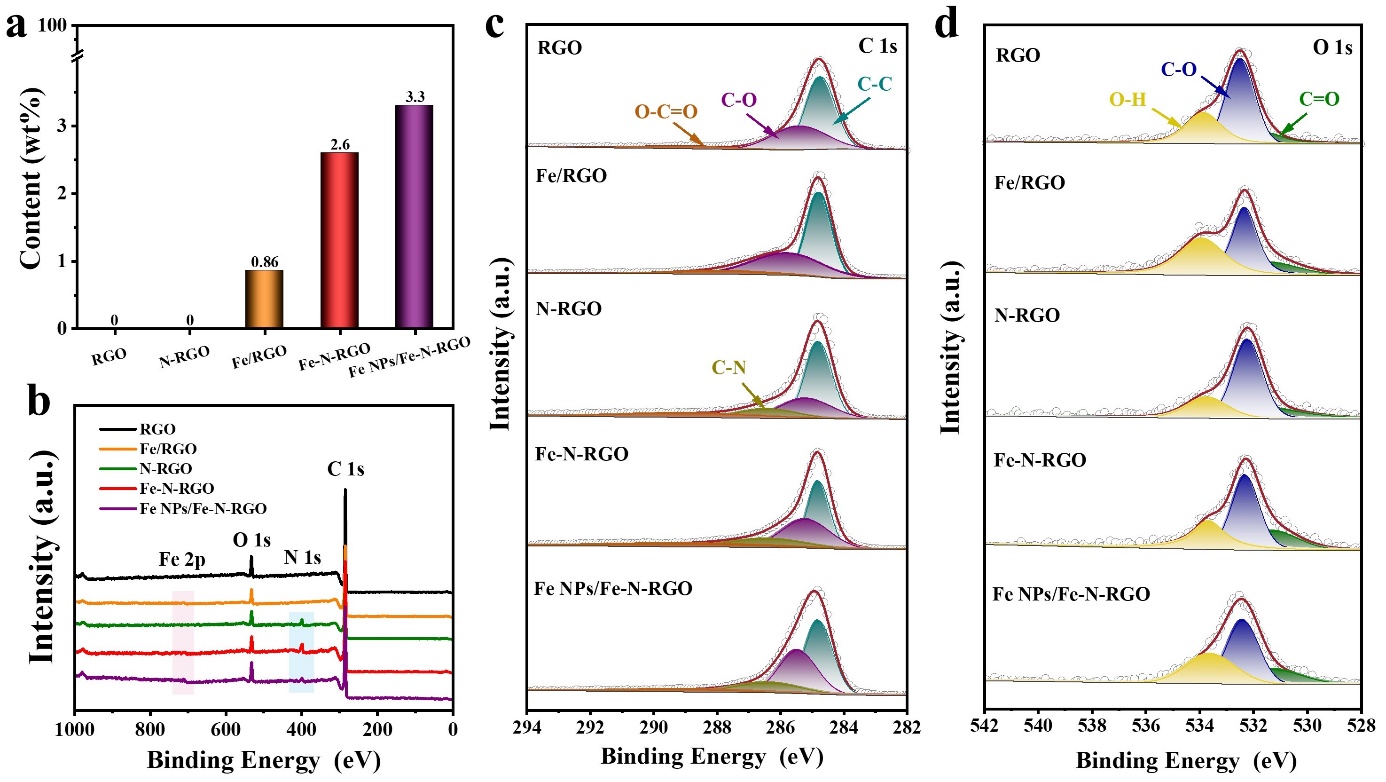
Fig. S2 a** Fe content in ICP measurement. **b** XPS survey, high-resolution **c** C 1s and **d** O 1s XPS spectrum of RGO, Fe/RGO, N-RGO, Fe-N-RGO and Fe NPs/Fe-N-RGO, respectively

**Fig. S3** FTIR spectra of RGO, Fe/RGO, N-RGO, Fe-N-RGO and Fe NPs/Fe-N-RGO, respectively

**Fig. S4** The corresponding EXAFS fitting curves of Fe foil **a, b** and FePc **c, d** at k space and R space, respectively

**Fig. S5 a, c** N_2_ adsorption-desorption isotherms and **b, d** the corresponding pore size distribution of N-RGO and Fe-N-RGO

**Fig. S6 a** Real part (µ') and **b** imaginary part (µ") of permeability of RGO, Fe/RGO, N-RGO, Fe-N-RGO and Fe NPs/Fe-N-RGO, respectively. **c** Magnetic hysteresis loops of Fe/RGO, Fe-N-RGO and Fe NPs/Fe-N-RGO

Due to the low Fe content (0.86 ~3.3 wt%) in Fe/RGO, Fe-N-RGO and Fe NPs/Fe-N-RGO, the μ' and μ" values of all samples are ≈1 and ≈0 (Fig. S5) with a low filler loading of only 1 wt%. The saturation magnetization (Ms) values are about 0, indicating that Fe has no obvious magnetic loss at the measured frequency in the alternating EM field.

**Fig. S7** RL curves with different thickness and frequency of **a** RGO, **b** Fe/RGO, **c** N-RGO, **d** Fe-N-RGO and **e** Fe NPs/Fe-N-RGO

**Fig. S8 a** σ and **b-f** Cole–Cole plots of RGO, Fe/RGO, N-RGO, Fe-N-RGO and Fe NPs/Fe-N-RGO

**Fig. S9** Impedance match (*Z)* of RGO, Fe/RGO, N-RGO, Fe-N-RGO and Fe NPs/Fe-N-RGO

The impedance matching ratio (*Z*) of samples can be calculated as:

$Z=|\frac{z_{r}}{z_{0}}|$ (S1)

$z_{r}=\sqrt{\frac{\mu_{r}}{\varepsilon_{r}}}, z_{0}=\sqrt{\frac{\mu_{0}}{\varepsilon_{0}}},$ (S2)

$Z_{r}$ represents the material′s intrinsic impedance and $Z_{0}$ is the free space impedance. The closer the *Z* value of the absorbing material is to 1, the better the impedance matching, so that more electromagnetic waves enter the material.

**Fig. S10** RL values, calculated and experimental values of matching thicknesses *t*_m_ under λ/4 condition, impedance matching values for **a** N-RGO and **b** Fe-N-RGO over 2-18 GHz

**Fig. S11 a** µ', **b** µ", **c** tanδ_μ_, **d** σ **e** ε_c_″ and **f** ε_p_″ of M-N-RGO composites (M= Mn, Fe, Co, Ni, Cu, Zn, Nb, Cd and Sn)

**Fig. S12** 2D color-mapping RLvalues with different thickness of Cu-N-RGO **a** and Zn-N-RGO **b**. **c** *Z* and **d** α of M-N-RGO composites (M= Mn, Fe, Co, Ni, Cu, Zn, Nb, Cd and Sn)

**Fig. S13** DOS and PDOS of M-N-Graphene composites (M= Mn, Fe, Co, Ni, Cu, Zn, Nb, Cd and Sn)

**Table S1** EXAFS fitting structural parameters at the Fe K-edge of Fe foil, FePc and Fe-N-RGO

| **Sample** | **Path** | **C.N.** | **R(Å)** | **σ^2^(Å^2^)** | **ΔE_0_ (eV)** | **R factor** |
| --- | --- | --- | --- | --- | --- | --- |
| Fe foil | Fe-Fe | 8* | 2.46±0.01 | 0.0053±0.0010 | 4.1±0.5 | 0.0092 |
|  | Fe-Fe | 6* | 2.85±0.01 | 0.0049±0.0011 | 4.6±0.8 |  |
| FePc | Fe-N | 4.0±0.4 | 1.97±0.01 | 0.0058±0.0017 | -0.2±0.5 | 0.0075 |
| Fe-N-RGO | Fe-N | 3.9±0.4 | 1.98±0.01 | 0.0162±0.0020 | -1.8±0.5 | 0.0032 |

C. N. is the coordination number; R is interatomic distance; σ^2^ is Debye-Waller factor (the Mean Square Relative Displacement (MSRD)); ΔE_0_ is inner potential correction. R factor is used to value the goodness of the fitting.

**Table S2** The optimal EMWA properties of reported N-doped RGO-based EM absorbers

| EM absorbers | Mass Ratio (wt%) | EAB | | RL | | Refs. |
| --- | --- | --- | --- | --- | --- | --- |
|  |  | EAB_max_ (GHz) | d (mm) | RL_min_ (dB) | d (mm) |  |
| N-rGO@CNTs | 2 | 7.1 | 2.6 | -49.4 | 2.6 | [S1] |
| CoP/NC@rGO | 4 | 7 | 2.71 | -67.5 | 2.14 | [S2] |
| N-doped-rGO/g-C_3_N_4_ | 5 | 4.56 | 1.6 | -49.59 | 1.6 | [S3] |
| AgNWs@NGA | 5 | 3.5 | 2.66 | -79.99 | 2.66 | [S4] |
| NGAs | 6 | 6.8 | 2.35 | -56.4 | 2.0 | [S5] |
| rGO/N-C/FeNi | 8 | 6.88 | 2.2 | -68.87 | 2.5 | [S6] |
| N-rGA/Ni | 10 | 5.1 | 2.1 | -60.8 | 2.1 | [S7] |
| NiO/NiFe_2_O_4_@N-rGA | 15 | 6.58 | 1.93 | -57.7 | 1.93 | [S8] |
| NRGO/CoFe_2_O_4_ | 15 | 5.2 | 1.8 | -44.7 | 1.8 | [S9] |
| CoFe_2_O_4_/N-rGO | 20 | 6.48 | 2.2 | -60.4 | 2.1 | [S10] |
| LaFeO_3_/N-rGO | 20 | 6.72 | 2.83 | -64.5 | 2.83 | [S11] |
| FeCo@NC/NCR/rGO | 20 | 5.28 | 1.66 | -59.42 | 1.42 | [S12] |
| NS-rGO/Fe_3_O_4_/C | 20 | 5.04 | 2.7 | -46.33 | 2.7 | [S13] |
| NRGO/MgFe_2_O_4_ | 25 | 4.8 | 2.0 | -48 | 2.0 | [S14] |
| ZnO/CuO/N-RGO | 25 | 2.8 | 2.8 | -17.1 | 2.8 | [S15] |
| Fe-Co/NC/rGO | 25 | 9.29 | 2.63 | -43.26 | 2.5 | [S16] |
| γ-Fe_2_O_3_@N-rGO/MWCNT | 25 | 3.4 | 3.5 | -59.2 | 4.98 | [S17] |
| Cu/NC@Co/NC | 35 | 5.19 | 2.5 | -54.13 | 3 | [S18] |
| CoFe@NC/rGO | 35 | 4.48 | 1.7 | -53.0 | 2.4 | [S19] |
| Fe_4_N/N-rGO | 50 | 5.27 | 1.61 | -53.1 | 1.6 | [S20] |
| FePc/N-rGO | 60 | 4.2 | 3.8 | -49.3 | 3.8 | [S21] |
| **N-RGO** | **1** | **6.41** | **2.16** | **-45.27** | **3.5** | **This work** |
| **Nb-N-RGO** | **1** | **4.96** | **2.19** | **-43.31** | **2.0** | **This work** |
| **Zn-N-RGO** | **1** | **5.16** | **2.16** | **-48.33** | **4.0** | **This work** |
| **Fe-N-RGO** | **1** | **7.05** | **1.89** | **-74.05** | **2.0** | **This work** |

**Supplementary References**

1. Z. Sun, Z. Yan, K. Yue, A. Li, L. Qian, Multi-scale structural nitrogen-doped rGO@CNTs composites with ultra-low loading towards microwave absorption. Appl. Surf. Sci. **538**, 147943 (2021). <https://doi.org/10.1016/j.apsusc.2020.147943>
2. G. Qin, X. Huang, Y. Liu, K. Zhang, Y. Yan et al., MOF-derived N-doped carbon nanocages trap CoP nanoparticles anchored on rGO to modulate dielectric polarization behavior for microwave absorption. Compos. Part A-Appl. S. **175**, 107808 (2023). <https://doi.org/10.1016/j.compositesa.2023.107808>
3. Q. Su, Y. He, D. Liu, K. Jia, L. Xia et al., Facile fabrication of ultra-light N-doped-rGO/g-C_3_N_4_ for broadband microwave absorption. J. Colloid Interf. Sci. **650**, 47-57 (2023). <https://doi.org/10.1016/j.jcis.2023.06.151>
4. X. Shu, S. Yan, B. Fang, Y. Song, Z. Zhao, A 3D multifunctional nitrogen-doped RGO-based aerogel with silver nanowires assisted self-supporting networks for enhanced electromagnetic wave absorption. Chem. Eng. J. **451**, 138825 (2023). <https://doi.org/10.1016/j.cej.2022.138825>
5. R. Shu, G. Zhang, C. Zhang, Y. Wu, J. Zhang, Nitrogen‐doping‐regulated electromagnetic wave absorption properties of ultralight three‐dimensional porous reduced graphene oxide aerogels. Adv. Electron. Mater. **7**, 2001001 (2020). <https://doi.org/10.1002/aelm.202001001>
6. H. Zhang, C. Shi, Z. Jia, X. Liu, B. Xu et al., FeNi nanoparticles embedded reduced graphene/nitrogen-doped carbon composites towards the ultra-wideband electromagnetic wave absorption. J. Colloid Interf. Sci. **584**, 382-394 (2021). <https://doi.org/10.1016/j.jcis.2020.09.122>
7. J. Tang, N. Liang, L. Wang, J. Li, G. Tian et al., Three-dimensional nitrogen-doped reduced graphene oxide aerogel decorated with Ni nanoparticles with tunable and unique microwave absorption. Carbon **152**, 575-586 (2019). <https://doi.org/10.1016/j.carbon.2019.06.049>
8. Q. Wang, J. Wang, Y. Zhao, Y. Zhao, J. Yan et al., NiO/NiFe_2_O_4_@N-doped reduced graphene oxide aerogel towards the wideband electromagnetic wave absorption: Experimental and theoretical study. Chem. Eng. J. **430**, 132814 (2022). <https://doi.org/10.1016/j.cej.2021.132814>
9. J. Xu, R. Shu, Z. Wan, J. Shi, Construction of three-dimensional hierarchical porous nitrogen-doped reduced graphene oxide/hollow cobalt ferrite composite aerogels toward highly efficient electromagnetic wave absorption. J. Mater. Sci. Technol. **132**, 193-200 (2023). <https://doi.org/10.1016/j.jmst.2022.05.050>
10. X. Wang, Y. Lu, T. Zhu, S. Chang, W. Wang, CoFe_2_O_4_/N-doped reduced graphene oxide aerogels for high-performance microwave absorption. Chem. Eng. J. **388**, 124317 (2020). <https://doi.org/10.1016/j.cej.2020.124317>
11. K. Fu, J. Zhao, F. Liu, L. Wu, Z. Jin et al., Enhanced electromagnetic wave absorption of nitrogen-doped reduced graphene oxide aerogels with LaFeO_3_ cluster modifications. Carbon **210**, 118071 (2023). <https://doi.org/10.1016/j.carbon.2023.118071>
12. J. Luo, H. Guo, J. Zhou, F. Guo, G. Liu et al., Rational construction of heterogeneous interfaces for bimetallic MOFs-derived/rGO composites towards optimizing the electromagnetic wave absorption. Chem. Eng. J. **429**, 132238 (2022). <https://doi.org/10.1016/j.cej.2021.132238>
13. K. Zhang, X. Gai, X. Zhang, X. Chen, H. L et al., Preparation of nitrogen and sulfur co‐doped graphene/Fe_3_O_4_/C nanocomposite and study on the absorbing properties. J. Mater. Sci-Mater. El. **32**, 8807-8818 (2021). <https://doi.org/10.1007/s10854-021-05552-y>
14. R. Shu, X. Li, Y. Wu, J. Zhang, Z. Wan, Fabrication of magnesium ferrite microspheres decorated nitrogen-doped reduced graphene oxide hybrid composite toward high-efficiency electromagnetic wave absorption. J. Alloy. Compd. **859**, 157865 (2021). <https://doi.org/10.1016/j.jallcom.2020.157865>
15. X. Liu, X. Lu, H. Guan, X. Liu, Y. Wang et al., Controllable synthesis of flower-like ZnO modified by CuO nanoparticles/N-RGO composites for efficient microwave absorption properties. Ceram. Int. **48**, 6948-6955 (2022). <https://doi.org/10.1016/j.ceramint.2021.11.251>
16. S. Wang, Y. Xu, R. Fu, H. Zhu, Q. Jiao et al., Rational construction of hierarchically porous Fe-Co/N-doped carbon/rGO composites for broadband microwave absorption. Nano-Micro Lett. **11**, 76 (2019). <https://doi.org/10.1007/s40820-019-0307-8>
17. W. Hou, Q. Liao, M. Wu, K. Liao, Y. Song et al., High-performance pinecone-like MOF derivative electromagnetic wave-absorbing composite via in situ anisotropic-oriented growth. J. Alloy. Compd. **937**, 168283 (2023). <https://doi.org/10.1016/j.jallcom.2022.168283>
18. H. Zhu, Q. Jiao, R. Fu, P. Su, C. Yang et al., Cu/NC@Co/NC composites derived from core-shell Cu-MOF@Co-MOF and their electromagnetic wave absorption properties. J. Colloid Interf. Sci. **613**, 182-193 (2022). <https://doi.org/10.1016/j.jcis.2021.11.166>
19. S. Wei, T. Chen, Z. Shi, S. Chen, Preparation of CoFe@N-doped C/rGO composites derived from CoFe prussian blue analogues for efficient microwave absorption. J. Colloid Interf. Sci. **610**, 395-406 (2022). <https://doi.org/10.1016/j.jcis.2021.12.051>
20. J. Ren, L. Zhang, N. Bao, X. Fan, H. Yang, Graphene-assisted synthesis of Fe_4_N with enhanced microwave absorption performance. J. Electron. Mater. **51**, 966-977 (2022). <https://doi.org/10.1007/s11664-021-09412-7>
21. J. Zhang, L. Chen, X. Li, H. Cao, W. Chen et al., Regulation dipole moments of N-doped graphene coordinated with FePc toward highly efficient microwave absorption performance in C band. Small 2308459 (2024). <https://doi.org/10.1002/smll.202308459>
